# Multivariable analysis of host amino acids in plasma and liver during infection of malaria parasite *Plasmodium yoelii*

**DOI:** 10.1186/1475-2875-12-19

**Published:** 2013-01-16

**Authors:** Erisha Saiki, Kenji Nagao, Hiroka Aonuma, Shinya Fukumoto, Xuenan Xuan, Makoto Bannai, Hirotaka Kanuka

**Affiliations:** 1 Department of Tropical Medicine, The Jikei University School of Medicine, 3-25-8, Nishi-Shinbashi, Minato-ku, Tokyo, 105-8461, Japan; 2 National Research Center for Protozoan Diseases, Obihiro University of Agriculture and Veterinary Medicine, Inada-cho, Obihiro, Hokkaido, 080-8555, Japan; 3 Institute for Innovation, Ajinomoto Co Inc, Kanagawa, 210-8680, Japan

**Keywords:** *Plasmodium yoelii*, Infection, Nutrition, Amino acid, Aminogram, Multivariable analysis

## Abstract

**Background:**

Malaria is the most significant human parasitic disease, and yet understanding of the energy metabolism of the principle pathogen, *Plasmodium falciparum*, remains to be fully elucidated. Amino acids were shown to be essential nutritional requirements since early times and much of the current knowledge of *Plasmodium* energy metabolism is based on early biochemical work, performed using basic analytical techniques, carried out almost exclusively on human plasma with considerable inter-individual variability.

**Methods:**

In order to further characterize the fate of amino acid metabolism in malaria parasite, multivariate analysis using statistical modelling of amino acid concentrations (aminogram) of plasma and liver were determined in host infected with rodent malaria parasite, *Plasmodium yoelii*.

**Results and conclusion:**

Comprehensive and statistical aminogram analysis revealed that *P. yoelii* infection caused drastic change of plasma and liver aminogram, and altered intra- and inter-correlation of amino acid concentration in plasma and liver. These findings of the interactions between amino acids and *Plasmodium* infection may provide insight to reveal the interaction between nutrients and parasites.

## Background

Malaria, the most significant human parasitic disease, remains a major cause of morbidity, anaemia, and mortality, in particular in developing countries. Malaria currently accounts for about two to three million deaths each year and estimates have been increasing over the last three decades [1]. It has long been acknowledged that populations residing in malaria-endemic areas generally live under conditions that lead to poor nutritional status. The groups at highest risk of the adverse effects of malaria, children and pregnant women, are also most affected by malnutrition. Infection by the human malaria parasite *Plasmodium falciparum* has severe and potentially lethal consequences for the metabolic state of the human host [2]. In all parasitic infections, there is a significant metabolic interaction between pathogen and host as the parasite diverts nutrients towards its own growth while the host exerts to maintain homeostasis. In the case of *P. falciparum* infection, the huge metabolic demands of the rapidly proliferating parasite cells, coupled with the effects of massive erythrocyte lysis, are responsible for the pathogenesis of the disease and its clinical manifestations, which include hypoglycaemia, lactic acidosis, haemolytic anaemia, haemoglobinuria and hypoargininaemia.

Despite the clinical and economic significance of *P. falciparum*, the energy metabolism of this organism is still poorly understood. Amino acids were shown to be essential nutritional requirements of the parasite a century ago [3]. For example, the severity of malaria is related to arginine concentration in plasma and L-arginine administration to cerebral malaria patients reverts endothelial dysfunction [4,5]. Extensive genomic and biochemical evidence indicate that many parts of parasite metabolism have been radically rationalized or modified. *Plasmodium* parasites are incapable of amino acid biosynthesis, relying on haemoglobin catabolism and uptake from the extracellular space [6]. Upon invading an erythrocyte, the parasite initiates a catabolic process whereby haemoglobin from the erythrocyte cytoplasm is ingested and proteolysed into its constituent amino acids in an acidic vacuole [7]. Therefore, the growth and multiplication of parasites are dependent on the presence in the extracellular solution of a set of amino acids, for example, isoleucine or methionine [8-11], that are absent or very little from adult human haemoglobin [12]. In addition, amino acids are substrate of proteins and play a central role as intermediates in nutrition metabolism, which form complicated networks, such as lipid, nitrogen and carbohydrate [13]. Thus, a novel analytic method is required to be developed in a blanket manner to understand the interaction among parasites and host amino acid metabolisms.

This study aimed to perform multivariable analysis using statistical modelling of amino acid concentrations (aminogram) [14,15] of plasma and liver of the host that was infected by rodent malaria parasite, *Plasmodium yoelii*. Plasma aminogram analysis indicates comprehensive survey of the amino acid concentrations in plasma, which has already been used in diagnosis of cancer [16]. Due to the importance of *Plasmodium* amino acid metabolism for malaria pathogenesis and as a target for most current candidate anti-malarial pharmaceuticals, it is critical to understand the dynamics of the amino acid metabolic networks in *Plasmodium* subspecies. Alternative approaches to reconstructing these networks, such as presumption from genomic data or *in vitro* biochemistry, are currently incomplete and metabolomics technologies are beginning to enable systems-level measurements of changes in metabolic activity [17,18]. This study provides a novel view to reveal the host-parasite interaction in aspects of nutrition dynamics, and the role played by these complicated pathways in maintaining the host-parasite balance.

## Methods

### Ethics statement

This study was carried out in strict accordance with the recommendations in the Guide for the Laboratory Animals of the Obihiro University of Agriculture and Veterinary Medicine and The Jikei University School of Medicine. The protocol was approved by the Committee on the Animal Experiments of the Obihiro University of Agriculture and Veterinary Medicine (Permit Number: 21–41 and 21–42) and The Jikei University School of Medicine (Permit Number: 23–020). All experiments using mice were performed under anaesthesia, and all efforts were made to minimize suffering in accordance with the Guidelines for Animal Experimentation of the Japanese Association for Laboratory Animal Science and the Fundamental Guidelines for Proper Conduct of Animal Experiment and Related Activities in Academic Research Institutions under the jurisdiction of the Ministry of Education, Culture, Sports, Science and Technology, Japan.

### Animals

Six- to eight-week-old male BALB/c mice (CLEA Japan) were infected with the *P. yoelii* 17XL strain by the intraperitoneal injection of infected blood. Asexual growth was monitored by Giemsa-stained smear. The animal room was maintained at a constant temperature (23±2°C) and humidity (55±10%) with a 12:12 hours light–dark cycle (lights on 0800–2000). Under diethylether anaesthesia, blood and liver were collected. The liver samples were immediately frozen and stored at −80°C until analysis.

### Parasite infection

Donor BALB/c mice were intraperitoneally injected with 1×10^6^ erythrocytes infected with *P. yoelii* 17XL strain [19,20]. Parasitaemia was monitored by counting the number of parasite-infected erythrocytes per 1,000 erythrocytes by microscopic examination of Giemsa-stained, thin (tail) blood smears.

### Amino acid analysis

Animals were bled under ether anesthesia; blood was collected into tubes containing EDTA as anticoagulant. After centrifugation, the supernatant was used for examination of plasma. Plasma samples were mixed with two volumes of 5% (w/w) trichloroacetic acid, and centrifuged (4°C, 20 min, 8,000 *g*) immediately after blood collection to remove precipitated protein. To prepare deproteinized tissue extracts, tissues were homogenized by using 5% trichloroacetic acid as described previously [21]. All samples were kept at 4°C during all steps to minimize chemical reactions of thiol metabolites. The amino acid concentrations were measured by an automatic amino acid analyzer (L-8800; Hitachi, Tokyo, Japan). Briefly, amino acids, separated by cation exchange chromatography, were spectrophotometrically detected after post-column reaction with ninhydrin reagent. Hydroxyproline content was determined by the method previously reported [22]. 20 amino acids (Asn, Gln, Asp, Thr, Ser, Glu, Pro, Gly, Ala, Cys, Val, Met, Ile, Leu, Tyr, Phe, His,, Lys, Trp, Arg), 11 their derivative (Hydroxyproline (Hypro), Hydroxylysine (Hylys), 3-methylhistidine (3-MeHis), 1-methylhistidine (1-MeHis), ethanolamine (EOHNH_2_), beta-alanine (beta-Ala), alpha-aminobutyric acid (alpha-ABA), Phosphoethanolamine (PEA), Taurine (Tau), Ornithine (Orn), Citrullline (Cit)), urea, and ammonia (NH_3_) were measured in this study.

### Aminogram analysis

Aminogram analysis was performed as previously described [14]. All data of plasma and liver concentrations of amino acids were analysed by using R (version 2.12.1; R foundation for statistical computing, Vienna, Austria [23]) for hierarchical cluster analysis. R was also used to construct coloured blocks in aminogram representing levels of amino acid concentrations, and to draw dendrogram using Ward's method [14]. The value for each amino acid concentration was normalized to a z score by using the mean and standard deviations of the scores.

### Spearman’s rank correlation coefficient analysis

Intra- or inter-correlations among amino acid concentrations in plasma and liver were tested by non-parametric equivalent Spearman’s rank correlation co-efficient analysis [24,25]. The calculations were performed with R, and the results of pair-wise correlations are shown as heat map (|ρ|≧0.7, *p*<0.05).

## Results

### Plasma and liver amino acid concentrations vary in host infected with *Plasmodium yoelii*

**Figure 1:**
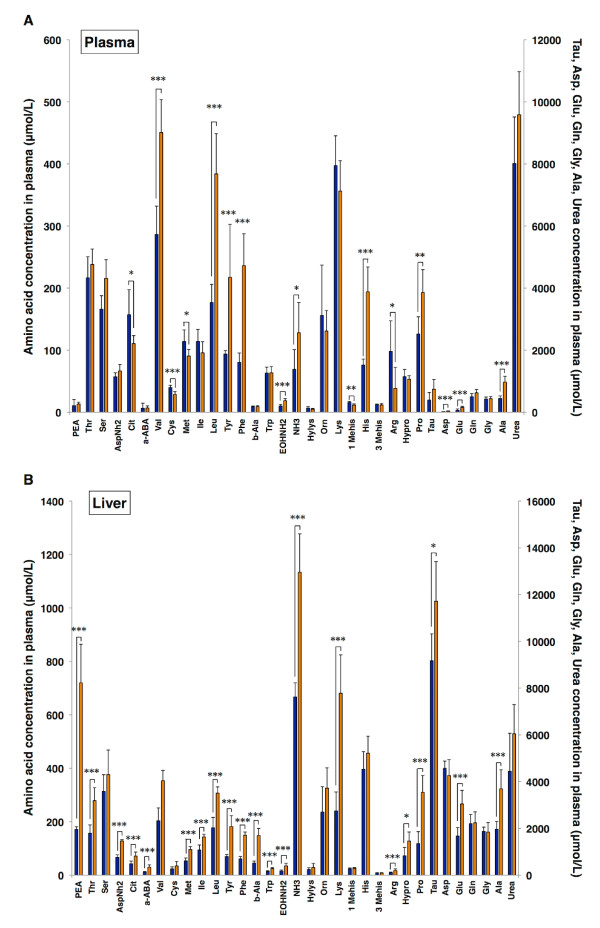
**Plasma and liver amino acid concentrations vary in host infected with *Plasmodium yoelii*.** Amino acid concentrations in plasma (**A)** and liver (**B**) were analysed on five days post-infection with *Plasmodium yoelii*. Shown are amino acids concentrations of uninfected mice (blue bars), infected mice (orange bars). Each bar represents mean ± SD of six mice. Asterisks denote: *p<0.05; **p<0.01; ***p<0.001. BALB/c mice were infected with 1×10^6^ RBCs parasitized with *P. yoelii*.

**Figure 2:**
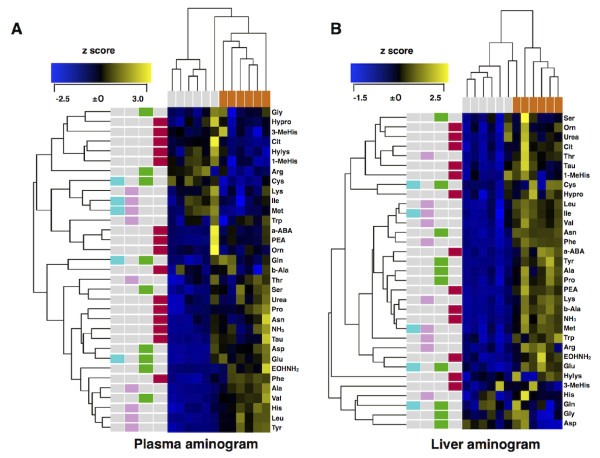
**Aminogram in plasma and liver changes with *Plasmodium *infection.** Alterations of aminogram in plasma (**A**) and liver (**B**) during *Plasmodium* infection are shown. The blue- and yellow-coloured cells represent z scores calculated for each amino acid [(observed mean - normal control mean)/normal control SD] in plasma and liver, respectively. Cluster analyses are applied to amino acids as described in the Methods. The z scores are as follows: yellow, positive changed; blue, negative changed; black, low variation. Coloured bars represent the mice condition and category of amino acids as follows; grey, uninfected mice; orange, *P. yoelii*-infected mice; light blue, essential amino acid for *P. falciparum*; pink, essential amino acid for mouse; green, non-essential amino acid for mouse; red, amino acid derivatives, urea, and ammonia.

To investigate amino acid metabolic profile in host during *Plasmodium* infection, concentrations of 20 amino acids, 11 their derivative, urea, and ammonia in plasma and liver were analysed in *P. yoelii*-infected mice whose parasitaemia was 84.5 ± 3.3%. Ten plasma amino acids (Val, Leu, Tyr, Phe, EOHNH_2_, His, Pro, Asp, Glu, Ala) and NH_3_ significantly increased, and five amino acids (Cit, Cys, Met, 1-MeHis, Arg) significantly decreased (Figure 1A). On the other hand, 21 liver amino acids (PEA, Thr, Asn, Cit, α-ABA, Met, Ile, Leu, Tyr, Phe, β-Ala, Trp, EOHNH_2_, Lys, Arg, HyPro, Pro, Tau, Glu, Ala) and NH_3_ significantly increased, and no amino acids significantly decreased (Figure 1B). Plasma and liver aminogram analysis based on heat-map [26] and cluster analysis [27] was then performed, demonstrating that individual plasma and liver aminogram were drastically altered by *P. yoelii* infection as well as forming infection-dependent cluster (Figures 2A and B). These data suggested that *Plasmodium* infection causes drastic alterations in host amino acid metabolism, potentially in a common pattern specific and characteristic of *Plasmodium* infection.

### Intracorrelation of aminogram variation in each tissue

**Figure 3:**
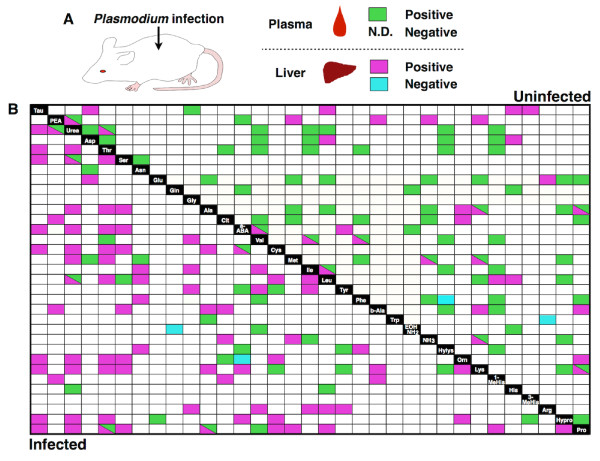
**Intracorrelation of aminogram variation in each tissue. **(**A**) A schematic diagram of the experiment to investigate intracorrelation of aminogram variation in plasma and liver. (**B**) Heat map of the correlation matrix among amino acids in plasma and liver. The plot summarizes the Spearman’s rank correlation coefficients between 20 amino acids, 11 their derivative, urea, and ammonia in plasma and liver of mice. Coloured panel represents significant and strong correlation (|ρ|≧0.7, *p*<0.05) between amino acid concentrations in plasma or liver of uninfected (upper) and infected (lower) mice. Each colour indicates as follows; green, positively correlated in plasma; magenta, positively correlated in liver; light blue, negatively correlated in liver; white, not correlated.

To better understand the relationship between *Plasmodium* parasites and host amino acid levels, multivariate-correlation analysis of amino acid concentrations in each of plasma and liver was performed. Spearman’s rank correlation co-efficient analysis was applied to plasma and liver samples from mice infected with *P. yoelii*. The scatterplots of 2 plasma amino acids show various patterns, such as positive and negative linear correlations or no correlations (data not shown). The results were summarized as heat-map based on Spearman’s rho and *p*-value (Figures 3A and B). In uninfected mice, the numbers of positively correlated pairs in amino acid concentrations were 67/528 (12.6%) in plasma and 31/528 (5.87%) in liver. In infected mice, the number of positively correlated pairs in plasma decreased to 28/528 (5.30%), while the number in liver increased to 81/528 (15.3%). Furthermore, Spearman’s test between parasitaemia and alteration of amino acid concentrations showed positive (Asp, Gly, His) and negative correlation (Tyr) (|ρ|≧0.7, *p*<0.05). These results indicated that *Plasmodium* infection induced alterations in intra-correlations of amino acid concentrations in each tissue.

### Inter-correlation of aminogram variation between plasma and liver

**Figure 4:**
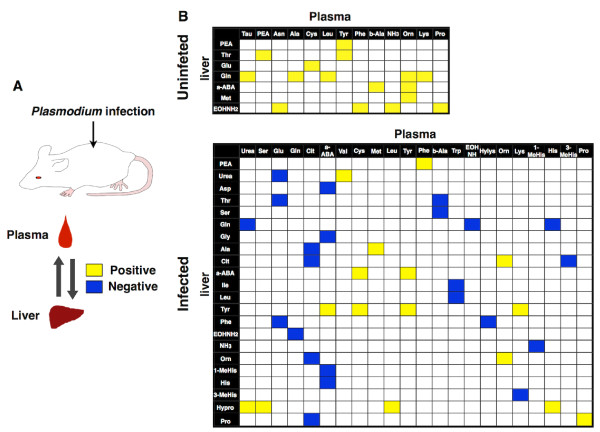
**Intercorrelation of aminogram variation between plasma and liver. **(**A**) A schematic diagram of the experiment to investigate intercorrelation of aminogram variation between plasma and liver. (**B**) Heat map of the correlation matrix among amino acids between plasma and liver. The plot summarizes the Spearman’s rank correlation coefficients among amino acids and their derivatives between plasma and liver. Coloured panel represents significant and strong correlation (|ρ|≧0.7, *p*<0.05) between plasma and liver amino acid concentrations in uninfected (upper) and infected (lower) mice. Each colour indicates as follows; yellow, positively correlated; blue, negatively correlated; white, not correlated.

The amino acid transportations among organs are intermediated by plasma. In order to acquire further information about the external interaction between plasma and liver, Spearman’s test for comparing correlations between plasma and liver amino acid concentrations was carried out. The number of positively correlated pairs in amino acid concentrations between plasma and liver were 16/1089 (1.47%) in uninfected mice, and 16/1089 (1.47%) in infected mice (Figures 4A and B). No pairs of negatively correlated were determined in uninfected mice, while pairs in infected mice were 23/1089 (2.11%). Interestingly, correlated pairs in uninfected mice did not match to any pairs in infected mice. These results indicated that parasite infection drastically changed relationships between plasma and liver amino acid concentrations.

## Discussion

Host-pathogen interactions in malaria rely on an exchange of nutrients, specifically amino acids, between erythrocyte and parasites. Amino acid concentrations of plasma were constantly kept in a set range to maintain amino acid homeostasis, although the results indicated that the concentrations are highly varied during *Plasmodium* infection (Figure 1A). The alterations of plasma amino acid concentrations by parasite infection are considered to be the results of export/import promotion of amino acids from/to erythrocytes, erythrocyte rupture, immunological enhancement, and acceleration of haematopoiesis. Since the changes of amino acid concentrations in liver influenced on plasma amino acid concentrations (Figure 1B), the variations of amino acid concentrations in other organs may also alter them. Furthermore, the enormous complexity of nutrition network makes it difficult to maintain amino acid homeostasis during parasite infection. Therefore, aminogram may lose the constancy among individual hosts during *Plasmodium* infection, although plasma and liver aminogram kept the potentially homogeneous pattern (Figures 2A and B). Amino acids are transported through blood, and their metabolism plays a central role in nutrient metabolisms; in other words, plasma aminogram indirectly reflects the metabolic dynamics in the host [28]. Consequently, these findings suggested specific patterns of nutrition dynamics responding to *Plasmodium* infection.

Metabolomic analysis gives us deeper understanding of *Plasmodium* biology and malaria patient disease state. Aminogram analysis revealed the variational aspects of host amino acid concentrations during *Plasmodium* infection, and the results suggested that altered aminogram develop novel relationships between amino acids. Furthermore, the results indicated that *Plasmodium* infection changed the intercorrelation of amino acids in plasma as well as in liver. Since amino acids act as growth regulation factors for parasites, it is reasonable to hypothesize that there is suitable plasma aminogram for parasite proliferation. These findings suggested that *Plasmodium* infection disrupted the original link and established a new one between amino acids (Figures 4A and B); that is, infection leads to novel amino acid dynamics. Meanwhile, *Plasmodium* parasites convert property of erythrocyte into adequate condition for themselves by exporting many proteins to plasma [29], such as degraded haemoglobin [30]. In fact, positive correlation was shown between plasma aminogram and amino acid composition of haemoglobin (Spearman’s ρ=0.46, *p*<0.05), proposing a new model that *Plasmodium* parasites adjust amino acid dynamics of host to an appropriate state for themselves.

In summary, this study demonstrated that aminogram analysis and multivariate-correlation analysis of plasma and liver amino acids were efficient, to reveal the complicated interaction between parasites and host metabolisms. Through these analyses, a view to renew the traditional concepts of interaction between parasites and host was obtained. The expansion from metagenome to metabolome [31], such as aminogram analysis, is a promising approach to characterize life phenomenon, to find new candidate anti-malarial pharmaceuticals or preventative measures, and to give fresh insights into malaria control.

## Competing interests

The authors declare that they have no competing interests.

## Authors’ contributions

ES and HK conceived the study and wrote the paper. ES, KN, and MB carried out the aminogram analysis. ES, HA, SF and XX contributed to the data analysis and discussion. All the authors read and approved the final manuscript. MB and HK supervised the study.
